# Enabling Big Geoscience Data Analytics with a Cloud-Based, MapReduce-Enabled and Service-Oriented Workflow Framework

**DOI:** 10.1371/journal.pone.0116781

**Published:** 2015-03-05

**Authors:** Zhenlong Li, Chaowei Yang, Baoxuan Jin, Manzhu Yu, Kai Liu, Min Sun, Matthew Zhan

**Affiliations:** 1 NSF Spatiotemporal Innovation Center, George Mason University, Fairfax, VA, United States of America; 2 Yunnan Provincial Geomatics Center, Yunnan Bureau of Surveying, Mapping, and GeoInformation, Kunming,Yunnan, China; 3 Department of Computer Science, University of Texas—Austin, Austin, Texas, United States of America; University of Vigo, SPAIN

## Abstract

Geoscience observations and model simulations are generating vast amounts of multi-dimensional data. Effectively analyzing these data are essential for geoscience studies. However, the tasks are challenging for geoscientists because processing the massive amount of data is both computing and data intensive in that data analytics requires complex procedures and multiple tools. To tackle these challenges, a scientific workflow framework is proposed for big geoscience data analytics. In this framework techniques are proposed by leveraging cloud computing, MapReduce, and Service Oriented Architecture (SOA). Specifically, HBase is adopted for storing and managing big geoscience data across distributed computers. MapReduce-based algorithm framework is developed to support parallel processing of geoscience data. And service-oriented workflow architecture is built for supporting on-demand complex data analytics in the cloud environment. A proof-of-concept prototype tests the performance of the framework. Results show that this innovative framework significantly improves the efficiency of big geoscience data analytics by reducing the data processing time as well as simplifying data analytical procedures for geoscientists.

## Introduction

Geoscience data are a core component driving geoscience advancement [1]. Understanding the Earth as a system requires a combination of observational data recorded by sensors and simulation data produced by numerical models [2]. Over the past half century human’s capability to explore the Earth system has been enhanced with the emergence of new computing, sensor and information technologies [3]. While the technological advancements accelerate collecting, simulating and sharing geoscience data, they also produce Big Data for geosciences from at least two aspects. First, massive amounts of multi-dimensional data recording various physical phenomena are taken by the sensors across the globe, and these data are accumulated rapidly with a daily increase rate of terabytes to petabytes [4]. For example the meteorological satellite Himawari-9 collects ∼3 terabytes data from space every day [5]. Second, supercomputers enable geoscientists to simulate Earth phenomena with finer spatiotemporal resolution and greater space and time coverage, producing large amounts of simulated geoscience data.

Effectively processing and analyzing big geoscience data are becoming critical to challenges such as climate change, natural disasters, diseases and other emergencies. However, the ever growing big geoscience data exceed the capacity of computing and data management technologies [6]. This is particularly true in climate science, which normally produces hundreds of terabytes of data in model simulations [2,7].

In this paper, we first take big climate data analytics as a case study to exemplify three challenges in big geoscience data processing and analyzing and then demonstrate how our proposed solution could address these challenges.

### 1.1 A Study Case: Climate Model Sensitivity

Climate change is one of the biggest contemporary concerns for humankind due to its broad impacts on society and ecosystems worldwide [8]. Information about future climate is critical for decision makers, such as agriculture planning, emergency preparedness, political negotiations and intelligence [9]. However, a major problem the decision makers face is that different climate models produce different projected climate scenarios due to unknown model uncertainties. Testing the sensitivity of input parameters of a climate model is a standard modeling practice for determining the model uncertainties [10]. To do this, perturbed physics ensembles (PPEs) run a model hundreds or thousands of times with different model input parameters, followed by analyses of the model output and input to identify which parameter is more sensitive to simulated climate changes (diagnostic).

Climate@Home (http://climateathome.com/climate@home) is a project initiated by NASA to advance climate modeling studies [11]. In this project to study the sensitivity of ModelE (http://www.giss.nasa.gov/tools/modelE/, global climate model developed by NASA), 300 ensemble model-runs (PPE-300) are required for each experiment, sweeping seven atmospheric parameters in each model-run input (Table 1). The simulation period is from December 1949 to January 1961 with a 4° x 5° spatial resolution and a monthly time resolution. Each model run generates ∼10 gigabytes data in four dimensions (3D space and 1D time) with 336 climatic variables and totally three terabytes of data for the PPE-300 experiment.

**Table 1:** Seven tested atmospheric parameters in the PPE-300 experiment.

Parameter	Definition	Range	Default
funio_denom	Affects fraction of time that clouds in a mixed-phase regime over ocean are ice or water	5–25	22
autoconv_mult	Multiplies rate of auto conversion of cloud condensate	0.5–2	1
radius_mult	Multiples effective radius of cloud droplets	0.5–2	1
ent_conf1	Entrainment coefficient for convective plume	0.1–0.9	0.3
ent_conf2	Entrainment coefficient for secondary convective plume	0.1—0.9	0.6
U00a	Relative humidity threshold for stratus cloud formation	0.4–0.8	0.6
U00b	Relative humidity multiplier for low clouds	0.9–2.5	2.3

To identify which of the 336 output variables are sensitive to the seven input parameters, the three terabytes model output is analyzed. Specifically, the following steps are taken:

**S1. Simulation**: Run ModelE 300 times sweeping seven input parameters;
**S2. Preprocess**: Convert model output (monthly .acc files) into NetCDF files, and combine monthly data to reduce the file numbers;
**S3. Management**: Store and manage the NetCDF files in a file system or database;
**S4. Process**: For each of the 336 variables in each of the 300 runs, calculate the annual global and 10-year mean.
**S5. Analysis**: Conduct linear regression analysis for each Parameter-Variable (P, V) pair (totally 336*7 pairs) using the 300 runs; and
**S6. Visualization**: Identify and plot the variables most affected by the parameters.

### 1.2 Challenges Posed by Geoscience Data Analytics

Geoscience data analytics poses three computing challenges as exemplified in the climate model sensitivity study case.

**C1. Big data or data intensity:** Storing, managing, and processing massive datasets are grand challenge in geosciences [12,13,51]. For example, one PPE-300 experiment produces 3 terabytes of climate data. A scalable data management framework is critical for managing these datasets. Furthermore, geoscience data analytics need to deal with heterogeneous data formats (e.g., array-based data, text files, and images), access distributed data sources, and share the result. Different data access protocols (e.g., FTP, HTTP) and data service standards (e.g., WCS, WFS, and OpenDAP) are normally involved in each step’s input/output. Hence, a mechanism to encapsulate these heterogeneities is essential.

**C2. Computing intensity:** Multi-dimensions and heterogeneous data structures are intrinsic characteristics of geoscience data [14]. Processing and analyzing these complex big data are computing intensive, requiring massive amounts of computing resources. In the case study, S4 is computing intensive given the terabytes of 4-D data. A parallelization-enabled algorithm is one key to accelerate these processes. Another computing intensive aspect is climate simulation (S1), where each model-run requires ∼5 days to simulate a single 10-year scenario. Traditional computing cannot finish the 300 model-runs with reasonable effort and time [15]. In addition, parallelization requires more resources since processing threads are running at the same time. Therefore, supplying adequate computing resources is another key to tackle the computing intensity challenge.

**C3. Procedure complexity:** Geoscience data analytics normally require complex steps with a specific sequence [16]. For example, the study case needs six steps (S1 to S6) from data generation (simulation) to visualization. A workflow platform tailored for handling these procedures is critical for managing, conducting and reusing the processes. In addition, conducting each step requires different tools, libraries and external processing services. To accomplish an analytics task, geoscientists normally need to discover appropriate tools/libraries, write their own programs/scripts and deal with Linux command lines. For example, S2 requires data format conversion tools, and S4 requires specific tools using libraries (e.g., NetCDF-Java, http://www.unidata.ucar.edu/software/thredds/current/netcdf-java/). And, for S5 and S6, scientists need to program using R script or other languages. A mechanism to integrate these heterogeneous tools and libraries is essential.

Cloud computing is a new computing paradigm characterized by its on-demand self-service, broad network access, resource pooling, rapid elasticity and measured service [17]. Cloud computing provides potential computing solutions to the management, discovery, access, and analytics of the big geoscience data for intuitive decision support [4].

In this paper, we explore the idea of building a scientific workflow framework using cloud computing as the fundamental infrastructure to tackle the aforementioned challenges. In this framework, methodologies are proposed for leveraging cloud computing, parallel computing and Service Oriented Architecture (SOA) as follows: HBase stores and manages big geoscience data across distributed computers; a MapReduce-based algorithm framework supports parallel processing of geoscience data; service-oriented workflow architecture supports on-demand complex data analytics; and the whole framework is implemented in the cloud environment. The remainder of this paper details the framework in the following sequence: Section 2 reviews relevant research; Section 3 details the methodologies; Section 4 introduces a proof-of-concept prototype and experimental results; and Section 5 draws conclusions and discusses future research.

## Related Work

In this section, some related work, fundamental technologies and background for the research are discussed.

### 2.1. Database Technologies for Managing Big Geoscience Data

Over the past decades, relational databases management systems (RDBMS) (e.g., Oracle) have been used to manage a variety of scientific data including that of the geosciences [18]. With RDBMS metadata are normally managed in a relational database while the actual data are stored in file systems. The data can be accessed by querying the database to find the reference (file location). While this approach takes advantage of the matured relational database technology, it is limited in terms of scalability and reliability since the data are normally archived in raw files. In fact the evolution of geoscience data has exceeded the capability of existing infrastructure for data access, archiving, analysis and mining [19,20].

To overcome the drawbacks of the traditional RDBMS, an emerging group of projects are addressing the multi-dimensional geoscience data utilizing distributed data management (e.g., Integrated Rule-Oriented Data Systems: http://irods.org/, Climate-G testbed [21], the Earth System Grid Federation [52]). These projects provide a grid-based framework to manage big geoscience data in a distributed environment. However, they do not draw support from cloud computing [22], so the resources and services can neither be initiated on demand nor meet the requirements of high scalability, availability and elastic of computing processes. In addition, these systems are normally complicated and bulky, making them hard to be adopted for other scientific research and applications.

NoSQL databases [23] provide a potential solution to the traditional RDBMS problems while offering flexibility to be tailored for various requirements. Over the past several years NoSQL databases have been used to store and manage big data in a distributed environment. Compared to traditional RDBMS, NoSQL database has the characteristics of schema-free, default replication support and simple API [24]. The most prevalent NoSQL databases such as HBase [25] and Cassandra [26] are based on a BigTable [27] schema. HBase, an open source distributed database running on top of Hadoop Distributed File System (HDFS), provides high scalability and reliability by storing data across a cluster of commodity hardware with automatic failover support. Studies to harness the power of HBase to manage big geoscience data include that of Liu et al. [24], who proposed a method to store massive imagery data in HBase by introducing two specific tables (“*HRasterTable*” and “*HRasterDataTable*”), and Chen et al. [28], who proposed a mechanism to effectively search and manage remote sensing images stored in HBase. Unfortunately, less research attention has been focused on leveraging HBase to handle big array-based multi-dimensional data (e.g., NetCDF or HDF).

To address this shortcoming, a data decomposition mechanism is proposed to manage multidimensional geoscience data with HBase in a scalable cloud computing environment.

### 2.2. Parallelization Technologies to Process Big Geoscience Data

Computation and data intensive geoscience analytics are becoming prevalent. To improve scalability and performance, parallelization technologies are essential [29]. Traditionally, most parallel applications achieve fine grained parallelism using message passing infrastructures such as PVM [30] and MPI [31] executed on computer clusters, super computers, or grid infrastructures [32]. While these infrastructures are efficient in performing computing intensive parallel applications, when the volumes of data increase, the overall performance decreases due to the inevitable data movement. This hampers the usage of MPI-based infrastructure in processing big geoscience data. In addition, these infrastructures normally have poor scalability and allocating resources is constrained by computational infrastructure.

MapReduce [33], a parallelization model initiated by Google, is a potential solution to address the big data challenges as it adopts a more data-centered approach to parallelize runtimes, moving computation to the data instead of the converse. This avoids the movement of large volume data across the network which impacts performance. Hadoop (http://hadoop.apache.org/) is an open source implementation of MapReduce and has been adopted in the geoscience research community [15,28,34].

Since Hadoop is designed to process unstructured data (e.g., texts, documents, and web pages), the array-based, multi-dimensional geoscience data cannot be digested by Hadoop. Studies have explored processing geoscience data in Hadoop. One approach converts binary-based dataset into text-based dataset. For example, Zhao et al. [35] converted NetCDF data into text-based CDL (http://www.unidata.ucar.edu/software/netcdf/docs/netcdf/CDL-Syntax.html) files to allow parallel access of massive NetCDF data using MapReduce. Although straightforward, this approach poses two issues: the transformation sacrifices the integrity and portability of the NetCDF data as well as increases the data management complexity; and the transformed data volume may increase by several times from its original volume. The other approach reorganizes and stores the original NetCDF dataset in Hadoop supported files (e.g., Sequence Files, http://wiki.apache.org/hadoop/SequenceFile). Duffy et al. [36] leveraged Hadoop MapReduce to process climate data by converting the dataset into Hadoop Sequence Files, eliminating the issues that occurred in the first approach. However, all records must be fully traversed to match records since no index or query is supported by Sequence File, reducing the performance as the number of records increases.

To address this problem, this paper explores a mechanism to store big geoscience data in HBase (see Section 2.1). Based on the proposed data decomposition mechanism, a MapReduce-enabled framework is introduced to support on-demand accessing and processing in parallel of big geoscience data.

### 2.3. Scientific Workflow for Geosciences

Scientific workflow serves as a problem-solving environment simplifying tasks by creating meaningful sub-tasks and combining to form executable data analysis pipelines [37]. Scientific workflow provides mechanisms to discover, share, analyze, and evaluate research tools [38,39] and is a significant element of geospatial cyberinfrastructure [37,40–42]. Provenance tracking provided by workflow systems enables geoscientists to determine the reliability of the data and service products and validate and reproduce scientific results in cyberinfrastructure [43].

There are several scientific workflow systems including Kepler [37], Taverna [44], Triana [45], Trident [46], and VisTrails [47]. These systems compose and schedule complex workflows on a distributed environment, such as clusters and Grids [32]. As a new computing infrastructure, cloud computing is a new approach for deploying and executing scientific workflows [16]. Preliminary studies to evaluate feasibility and performance of migrating scientific workflows into the cloud [48–50] have found that cloud computing provides comparable performance with better scalability and flexibility to traditional computing infrastructure given similar resources. However, these studies mainly focused on deploying current scientific workflow platforms to the cloud environment by replacing traditional physical machines with virtual machines in existing workflow deployment. A more comprehensive study is desired to fully leverage the advantages of cloud computing to enable scientific workflow for supporting geoscience.

We propose a cloud-based workflow framework by incorporating cloud computing to provision on-demand the whole workflow execution environment, adding dynamically computing resources to the workflow during runtime and integrating heterogeneous tools seamlessly.

## Methodologies

### 3.1. Framework

**Fig 1:**
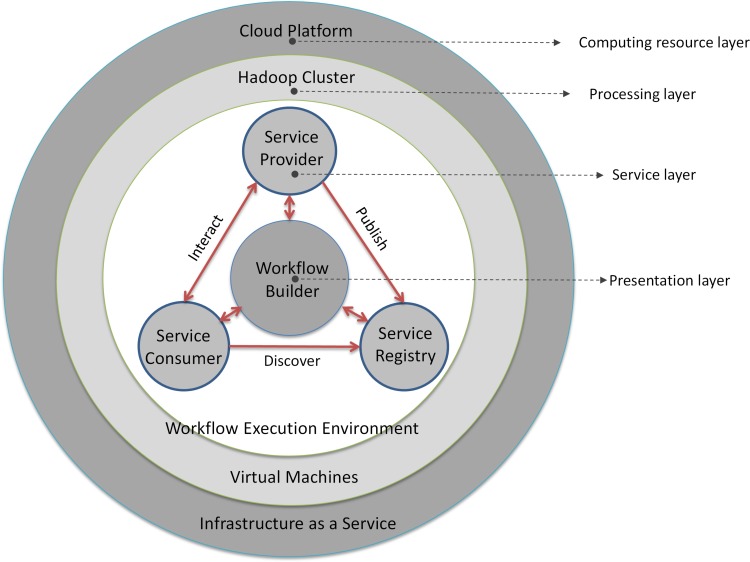
Framework architecture.

The framework (Fig. 1) is layer-based and includes four layers: computing resource (Cloud Platform); processing (Hadoop Cluster); service; and presentation (Workflow Builder). Cloud platform provides the on-demand computing resources including computing, storage, and network as services. The cloud platform includes a processing layer where the workflow engine running on the virtualized Hadoop cluster (virtual machines as cluster nodes). The service layer is built on top of the cluster for registering, managing, and chaining the services. The services are chained as executable workflows in an on-demand and scalable computing environment. Processing layer and service layer form the workflow execution environment. On top is the presentation layer which enables users to publish, discover and use services to build workflows in a drag-and-drop style, and runs and monitors workflows in a web-based interface. Oozie (Oozie http://yahoo.github.com/oozie/) is adapted as the workflow engine due to its intrinsic integration with Hadoop MapReduce.

In this framework, data intensity is handled through storing and managing data using HBase in a distributed environment. Computing intensity is tackled by allocating the intensive computation tasks to many computing nodes using the MapReduce model. By integrating cloud computing, computing resources associated with the workflow are provisioned or terminated on-demand to ensure performance while minimizing resource consumption.

Service Oriented Architecture (SOA) is adopted to publish different processes as individual services, and these not only include processing and data services but also offer infrastructure and tool services. Different from the traditional web services orchestration [55–56], the “service” herein does not refer to the “web service” but rather to a self-described functional unit plugged into the workflow. This section details the framework from big geoscience data process, service-oriented approach, and cloud-based workflow execution environment.

### 3.2. Big Geoscience Data Processing with MapReduce

#### 3.2.1. Multi-Dimensional Geoscience Data Decomposition Mechanism

This section details the mechanism to decompose the array-based data files and store them in HBase.

Normally, geoscience data are five dimensional: space (latitude, longitude, and altitude), time and variable. For the array-based data models, data are stored in individual files, regarded as a dataset. The dataset is located by the dataset id (e.g., file URI). The array-based data model is expressed as Equation 1, and each dataset id refers to a dataset containing five dimensions (X, Y, Z, T and V).

f(D)=DS(X,Y,Z,T,V) Equation 1

Where *DS = Dataset, V = Variable, T = Time, X = Latitude, Y = Longitude, Z = Altitude, and D = Dataset Id*

In HBase a straightforward way to store the array-based data is using *Dataset Id* as the row key and *Dataset* as row value. While this works for storing data, the parallelization of data processing is problematic because one dataset may reach gigabytes.

**Fig 2:**
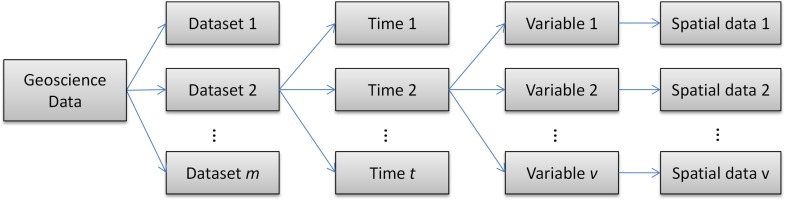
Hierarchical structure of the multi-dimensional geoscience data.

Based on the array-based data model, geoscience data is decomposed hierarchically (Fig. 2). Each dataset contains one or multiple timestamps, and at each timestamp there are multiple variables; each variable refers to a 2D or 3D data grid. Assuming each data grid as an *AtomDataset*, the decomposed data model is expressed as Equation 2

f(D,T,V)=ADS(X,Y,Z) Equation 2

Where *ADS = AtomDataset, V = Variable, T = Time, X = Latitude, Y = Longitude, Z = Altitude*, and *D = Dataset Id*.

Compared with Equation 1, the decomposed data model moves two dimensions T and V from the right to the left side. This triggers two changes: 5D dataset (X, Y, Z, T, V) is degraded to 3D *AtomDataset* (X, Y, Z) and single dataset id (D) becomes composite id(D, T, V). With this decomposition, large volumes of geoscience data are managed in a Bigtable style [27], where the (D, T, V) are stored as the composite row key and the *AtomDataset* as the row value in HBase.

Besides the scalability and reliability of HBase, this decomposition has three advantages. First, the D, T, and V are stored in HBase as columns in series enabling flexible search against the time, variable and dataset. Once data are loaded into HBase, the *AtomDataset* queries and accesses data through various filters. Second, new data can be seamlessly appended and integrated to the database without breaking current data structure. And third, parallelization with MapReduce algorithm is achieved in a finer granularity by decomposing the data from 5 to 3 dimensions.

#### 3.2.2. MapReduce-enabled Framework for Processing Big Geoscience Data

**Fig 3:**
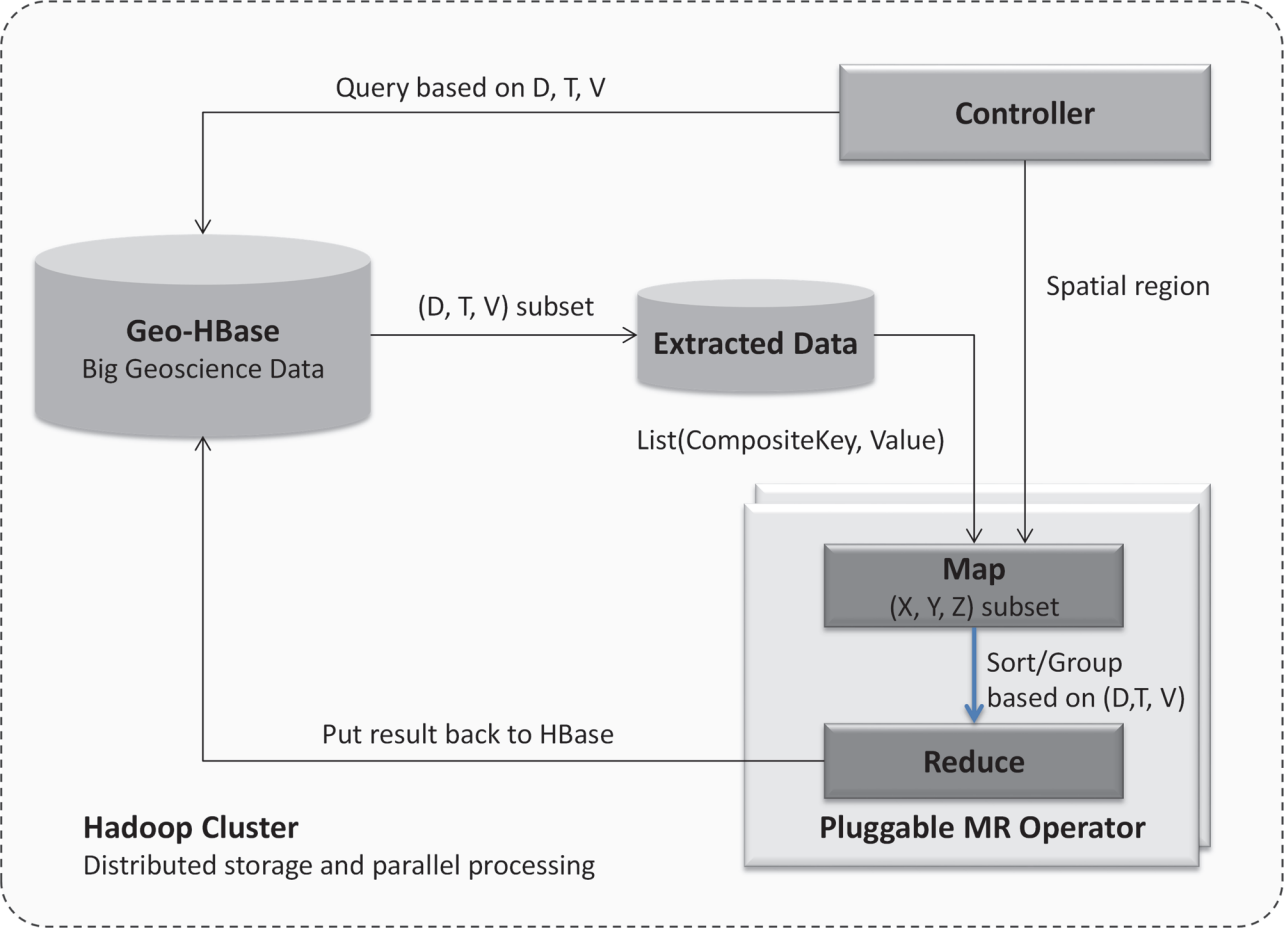
MapReduce-based framework for processing big geoscience data.

Based on the above data decomposition mechanism, we introduce a MapReduce-enabled framework to process big geoscience data. The back end of the framework is a Hadoop cluster deployed in the cloud environment that provides distributed storage and computing power. The framework contains the following components: *Geo-HBase, Controller*, and *Pluggable MR Operator* (Fig. 3).

- *Geo-HBase* stores the decomposed big geoscience data (Section 3.2.1). *Geo-HBase* supports flexible queries to the data repository based on dataset id, time, and variable, so a subset of interested data is effectively extracted and processed.
- *Pluggable MR Operator* is a MapReduce program conducting a processing task against the data stored in HBase (e.g., calculating an annual mean for selected variables, sub-setting the data based on user specified regions).
- *Controller* is the user interface allowing users to interact with the framework, such as starting a processing job with specified parameters.

A typical workflow for processing data with this framework is the following sequence: *Controller* sends processing request with the query parameters (dataset ids, time period, and variables) and spatial region; *Geo-HBase* extracts the required data based on the dataset id (D), time (T), and variable (V); the extracted data are loaded to *MR Operator* as a list of key-value pairs; the *Map* first conducts spatial (X, Y, Z) sub-setting based on the specified spatial region. The composite key sorts and groups the emitted intermediate data from *Map* based on the composition of (D, T, V) by *MR Operator*; and finally the result is written back to HBase.

**Fig 4:**
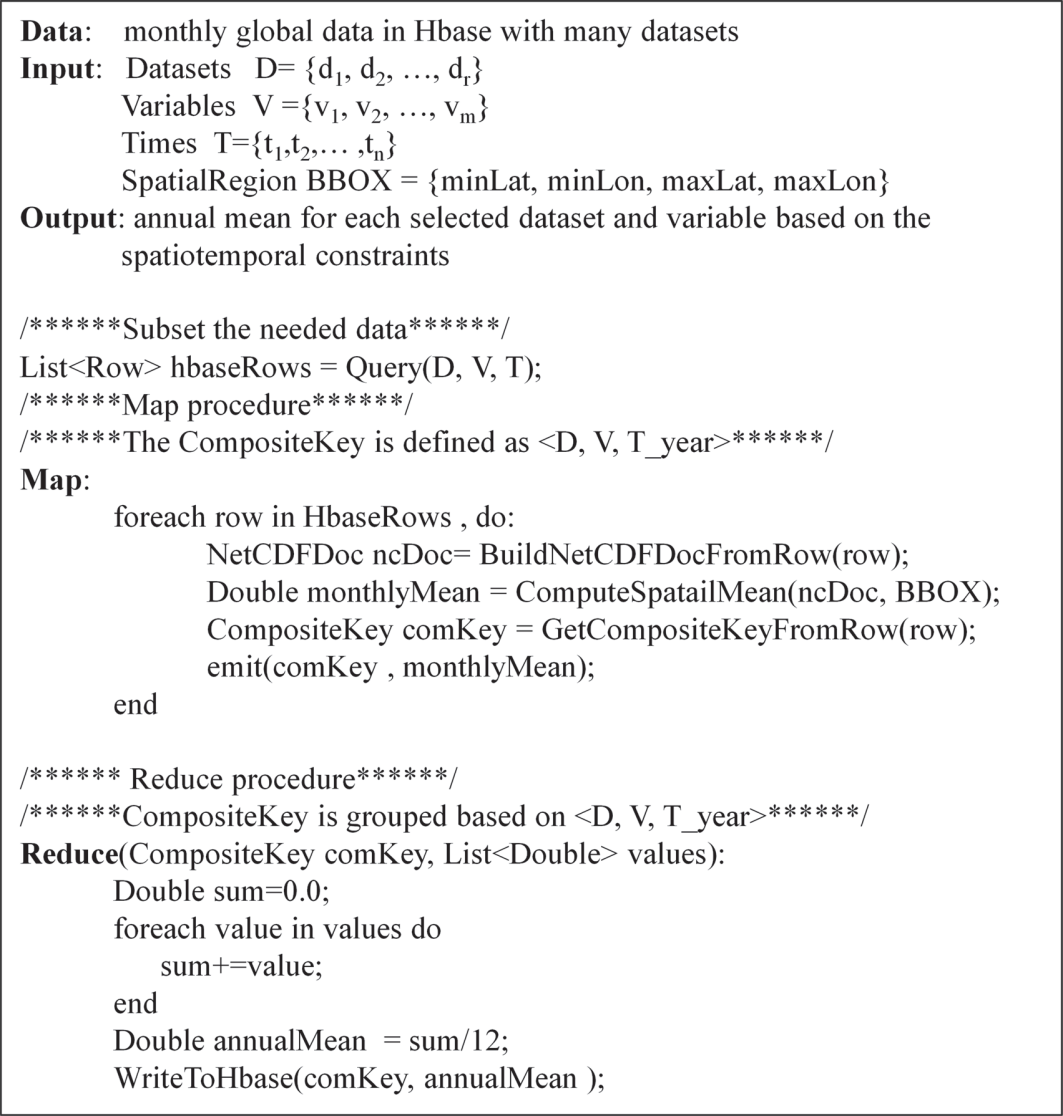
Algorithm for computing annual global mean of multiple datasets based on the framework.

Scientists can develop different MapReduce algorithms to process the data stored in Geo-HBase as *Pluggable MR Operators*. Furthermore, these *Pluggable MR Operators* are published as *Processing Services* that are used to build the workflow. Fig. 4 is an example MapReduce algorithm for calculating annual global mean of a subset of climate data.

### 3.3. Service-oriented Approach

#### 3.3.1. Service Model

The key to leveraging service-oriented concept in the workflow architecture is that each step in the workflow is abstracted as a service, and various services are chained to form a workflow. To ensure that different services can be connected in a unified fashion, we abstract each service as a processing unit with two general interfaces: input and output. For input, two types are defined: Input Parameter (IP) and Input Data (ID). Similarly there are two output types: Output Parameter (OP) and Output Data (OD). The input and output parameters are primitive, (e.g., numbers, short texts), whereas the input and output data refer to data files stored in the shared file system. Based on this, we define a unified service model as Expression 1, where the output data and parameter of one service are used as the input data and parameter by another service, thus enabling the servicing chaining.

$$Service(ID,IP)\overset{execute}{\to }(OD,OP)$$ Expression 1

Unlike traditional scientific workflow in which each step is normally the computational process, we define four types of services to build a workflow, and each is described below.

- **Processing Service** processes, analyzes or visualizes input data. Three types of programs are published as a processing service: MapReduce program processing the big geoscience data stored in HBase (Section 3.2); Java executable program conducting general processing task; and Shell script conducting data preprocess, statistics or visualization. For example, a Shell script calling R script to plot a climate variable is published as a processing service.
- **Data Service** focuses on fetching data from outside of the workflow as service input and publishing output as various services to share (Section 3.3.2).
- **Model Service** runs geoscience models (e.g., climate model) with user specified model input; the modeling environment of software configuration and computing resources running the model are automatically provisioned in the cloud [53].
- **Infrastructure Service** provisions the virtual machine-based services by leveraging the IaaS. Three types are included: provisioning pure computing resources (e.g., bare-metal virtual machine); provisioning computing platforms (e.g. Hadoop or MPI-based cluster); and provisioning virtual machines with pre-installed software packages or applications (e.g., virtual machine with R environment).

**Fig 5:**
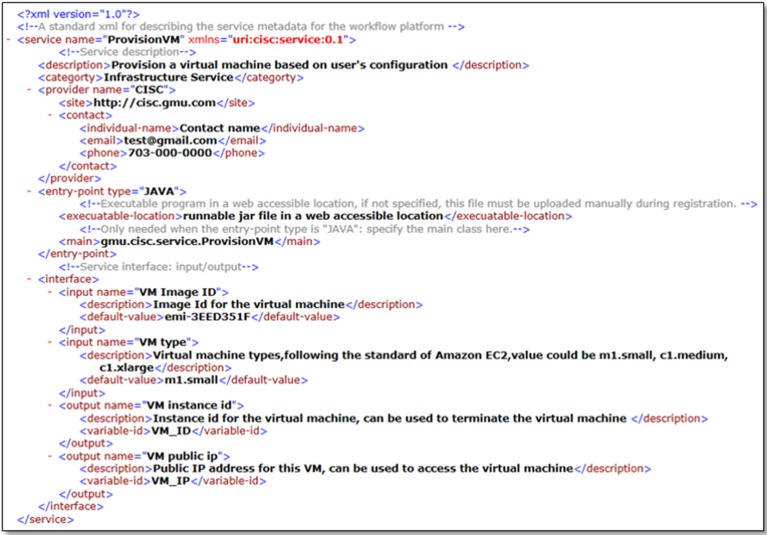
Service definition metadata example (Infrastructure Service: ProvisionVM). Please refer to the Supporting Information file (service definition XML schema.xsd) for the service metadata XML schema.

Following the service model, each service is composed of service executable program and service definition metadata. Service definition metadata is an XML describing the services (Fig. 5) and is comprised of three sections: service description of the general service information; service entry point indicating the location of the service executable program; and service interface detailing the service input and output along with semantic description. To register a service into the workflow framework, the service definition metadata is first interpreted to add the service in the service catalogue, and the service executable program is uploaded to the workflow execution environment.

#### 3.3.2. Loosely-coupled Service I/O Mechanism

**Fig 6:**
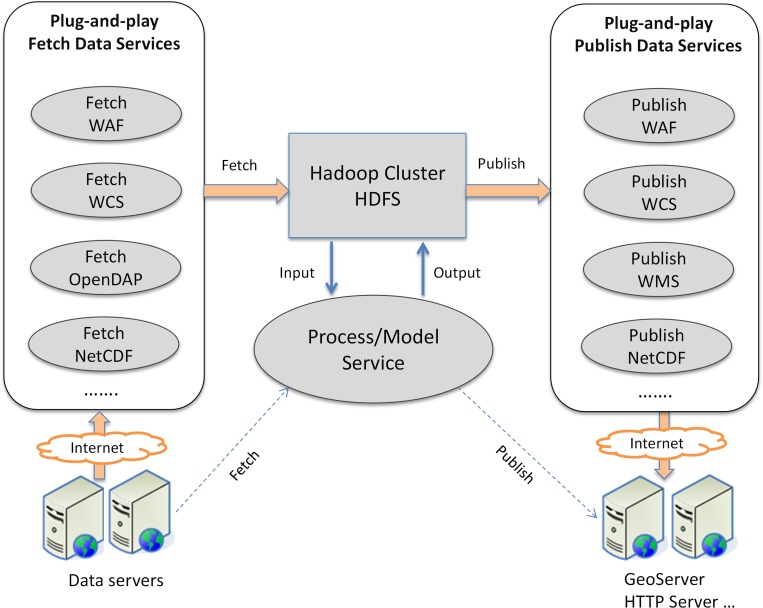
Loosely-coupled Service I/O mechanism.

The workflow engine is deployed on Hadoop, and the workflow tasks (services) are executed on different machines. Hence, it is important that all services read input and write output data in a shared file system to avoid extra data transfer loads. The HDFS is used as such a file system in the framework, providing a unified service execution environment. However, geoscience analytics often requires small to midsized data from remote data services (e.g., WFS, WCS, and OPenDAP) as part of the input, and publish the output as web services (e.g., WMS, WFS). One solution is that the service includes the function to fetch and publish data from remote services. However, at least two problems arise. The first is that data handling is tightly coupled with the processing logic, which makes it difficult for the service to incorporate other types of data services. The second is that each service implements its own data handling function which cannot be reused. We propose a loosely-coupled, service Input/output (I/O) mechanism as illustrated in Fig. 6 to address these shortcomings.

This mechanism extracts the data handling components and publishes them as individual workflow *Data Services*, including two categories: *Fetch Data Services* and *Publish Data Services. Fetch Data Service* fetches data from remote data servers and loads them into HDFS. Other services, such as *Processing Service* and *Model Service*, can access the data directly from HDFS. For example, *Fetch WAF* (Web Access Folder) service downloads data from a WAF and loads them to HDFS; *Fetch OpenDAP* service subsets data from an OpenDAP server. *Publish Data Service* requires a server to host the data. For example, to publish a *Processing Service’s* output as WMS, a WMS server (e.g., GeoServer) is required to host the service, and, an *Infrastructure Service* can be integrated into the workflow to provision a virtual machine with pre-installed GeoServer.

**Fig 7:**
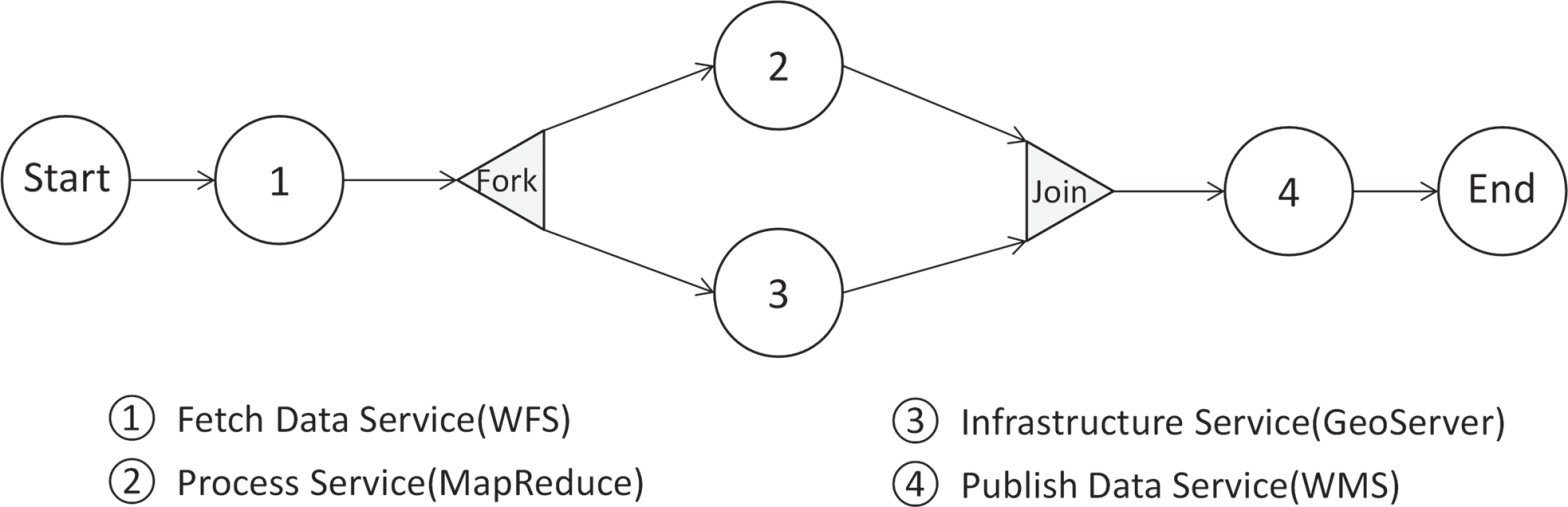
A typical workflow with four types of services.

Fig. 7 shows a typical workflow consisting of four different services:

1. A *Fetch Data Service* fetches vector data (U.S. state boundary) from a WFS server as the input of the *Processing Service*;
2. The *Processing Service* is a MapReduce program which calculates the monthly mean land surface temperature from the climate data stored in *Geo-HBase* using the boundary data as the statistics unit;
3. Meanwhile, an *Infrastructure Service* provisions a virtual machine with pre-installed GeoServer from the cloud platform; and
4. *Publish Data Service* publishes output data from process service to GeoServer as WMS.

This service I/O mechanism is flexible and extendable in that external services are supported by developing corresponding data services in the workflow platform. Once a data service is registered, it can be used by any other services to fetch/publish input/output. This service I/O mechanism addresses the challenge of heterogeneous and distributed data associated with each step’s input and output in the workflow.

### 3.4. Cloud-based Workflow Execution Environment

Scientific workflows normally require a collection of software components such as tools, libraries, programs, models, and applications, and these components are developed at different times by different people [16]. In this case study the workflow needs to setup and run a climate model, first with NetCDF Operator (NCO) library to preprocess the model output, followed by Hadoop MapReduce to parallel process model output, and then fed to a Java program (or R script) to conduct linear regression analysis and visualization. These heterogeneous software components must be seamlessly integrated into a coherent workflow. To achieve this, a traditional workflow system needs to pre-install the required software components on the physical machine (s), and this poses two problems. First, if the execution environment is backed by a cluster, the same software components must be configured on each machine, and any update to the execution environment is time consuming. Second, some software components are complex requiring specific execution environments that cannot be installed on the common environment. To address these shortcomings, we propose the workflow in the cloud environment with two mechanisms.

The first mechanism deploys the whole Workflow Execution Environment (WEE, Hadoop cluster) in the cloud. The entire WEE is “burned” to image, including Hadoop software, workflow engine, and library environment for executing the workflow tasks (e.g., R, NCO, JRE) and can be provisioned within minutes. The VMs are provisioned as cluster nodes based on the VM image (a snapshot of pre-configured operating system used to launch a VM). When an update is required, the VM image is re-built by installing new or removing old software components, and the WEE is re-provisioned quickly based on the new VM image. Another advantage is that new computing resources can be easily added to the WEE by provisioning more cluster nodes.

The second mechanism integrates specified software into VM images and publishes these images as *Infrastructure Services*. This is more flexible in that the software environment is self-contained and exposed as a standard infrastructure service in the workflow platform. These services are added and removed without affecting current WEE. In addition, the complex software components (e.g., climate model, GeoServer) are difficult to integrate into WEE due to the specified system requirement and high resource occupation, and publishing them as *Infrastructure Services* improves the system performance and flexibility. Furthermore, this mechanism provides an alternative to integrating legacy software that requires a specific execution environment into the workflow. Finally, the image-based *Infrastructure Service* offers a reproducible environment for certain tasks in the workflow.

## Prototype and Experiment Result

To verify the performance of the proposed framework, a proof-of-concept is offered, and an experiment is conducted for the aforementioned case study using the prototype.

### 4.1. Prototype Based on the Framework

#### 4.1.1. Cloud Environment Setup

The proposed framework is based on both private and public clouds. A private cloud platform on Eucalyptus (http://www.eucalyptus.com) 4.0 is established in our data center, serving as the cloud environment, and this selection is based on our previous study [54]. In addition, Eucalyptus has compatible Application Programming Interfaces (APIs) with Amazon’s Elastic Compute Cloud (Amazon EC2, http://aws.amazon.com/ec2/), a widely used public cloud service. The underlying hardware consists of 16 physical machines connected with 1 Gigabit Ethernet (Gbps), and each has an 8-core CPU running at 2.35 GHz with 16 GB of RAM and 60 GB of on-board storage. Totally, 120 m1.small VMs (1 core CPU running at 1 GHz and 2G of RAM) is provisioned in the cloud.

#### 4.1.2. Prototype Implementation

**Fig 8:**
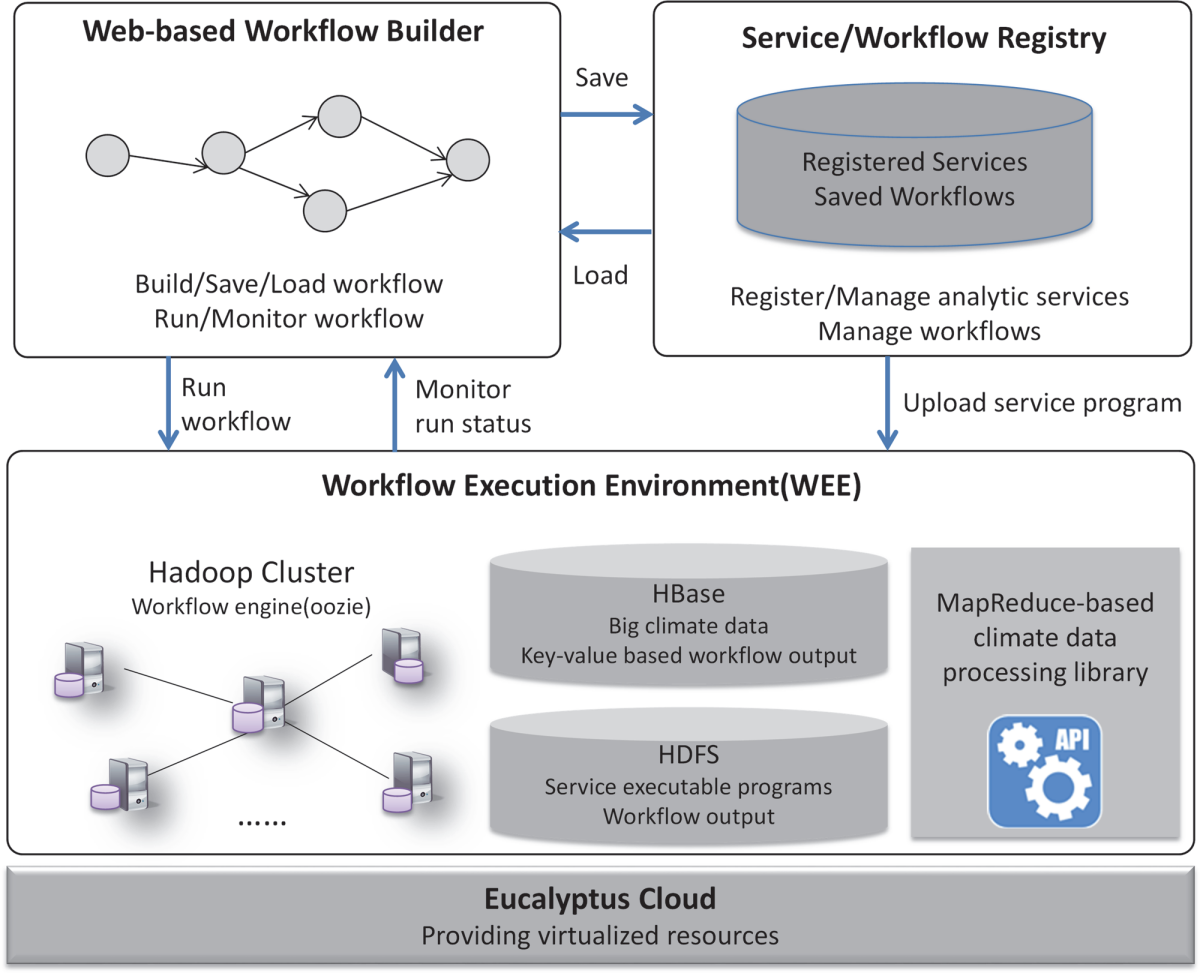
Prototype implementation architecture.

The prototype implementation architecture (Fig. 8) contains four major components: *Eucalyptus Cloud, Workflow Execution Environment (WEE), Web-based Workflow Builder*, and *Service/Workflow Registry*.

*Eucalyptus Cloud* provides virtualized computing resources. The WEE, built on top of the cloud platform, consists of computing, storage and processing libraries. The computing is provided by a virtualized Hadoop cluster and coordinated by the workflow engine (powered by Oozie). Storage is provided by HBase and HDFS, where HBase stores the decomposed big climate data and key/value-based workflow output, whereas HDFS stores the service executable programs and other workflow output.

*Service/Workflow Registry* is the service layer providing a database for managing the registered services and saved workflows. Service definition metadata (XML) and workflow definition files (XML) are stored in the database. During service registration, the service executable program is uploaded to WEE.

**Fig 9:**
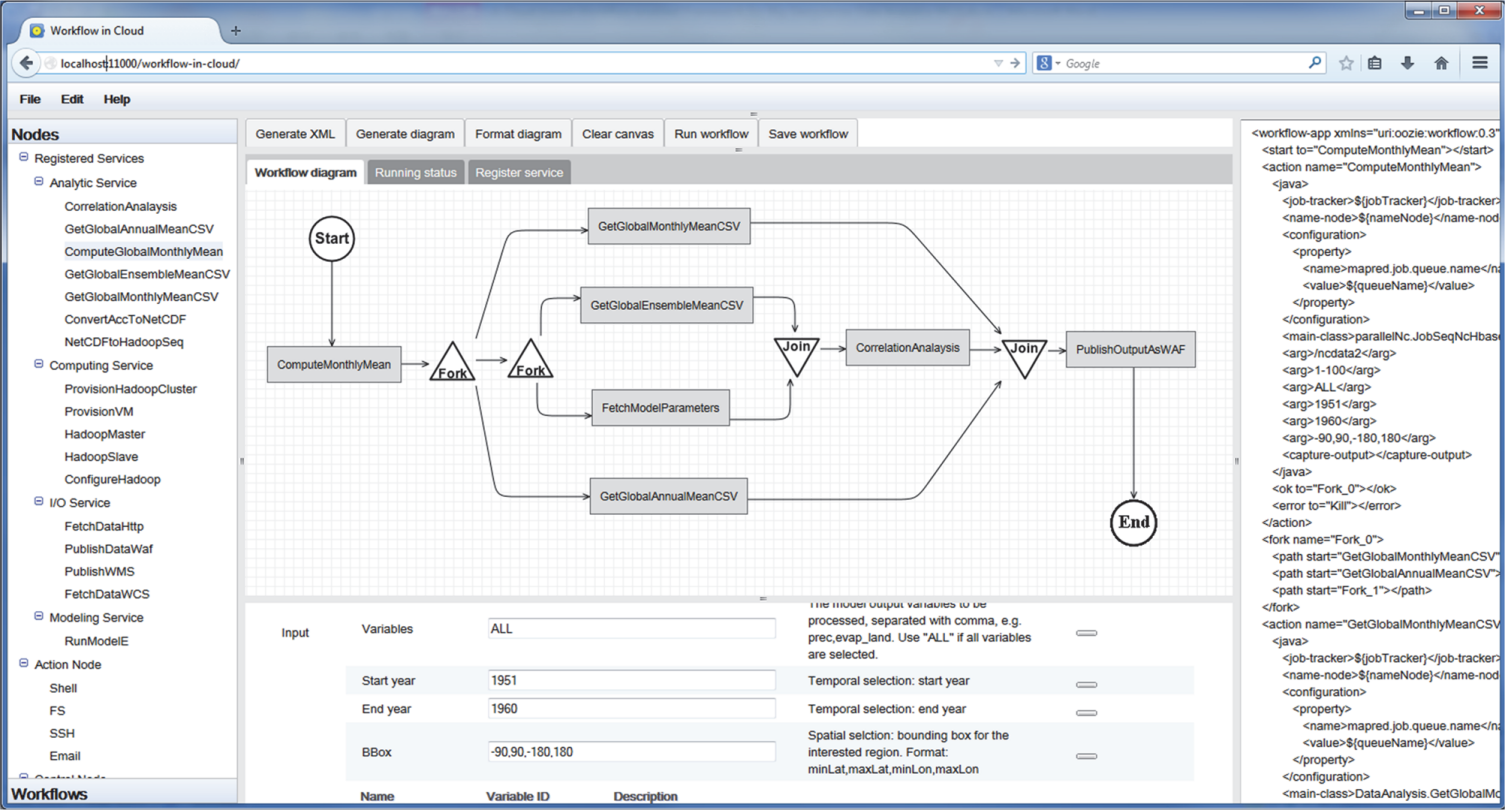
GUI of the web-based workflow builder.

*Web-based Workflow Builder* is the graphic interface (Fig. 9) through which users build workflow by visually connecting various services, run workflow by submitting the request to WEE with one-click, and monitor the workflow execution status in real time. Services and workflows are loaded to the builder from the registry. The workflow is saved to the server for re-running or downloaded as XML for sharing. The builder is based on the open source workflow-generator tool (https://github.com/jabirahmed/oozie/tree/master/workflowgenerator).

### 4.2. Experiment Result

#### 4.2.1. Executable Workflow for the Study Case

To demonstrate how the proposed workflow framework addresses the challenges posed by the study case (Section 1.2), over ten services are developed following the proposed service model. These are registered to the prototype system to facilitate the six steps of the study case.

For the S1, a *Model Service* (*RunModelE*) is based on our previous study [53] to setup and run ModelE automatically. This is also an *Infrastructure Service* since it provisions a virtual cluster with configured modeling environment to run the model. For S2, two *Processing Services* are developed with *AccToNetCDF* being a script-based service converting model out .acc files to NetCDF format, and *NetCDFtoHBase* using NCO library to decompose (split) the NetCDF and subsequently uploading into database using HBase APIs. For S4, a MapReduce-enabled *Processing Service* computes the global monthly mean for all model output. Finally, for S5 and S6 a Java-based *Processing Service* conducts linear regression analysis and plots the relationships for the most affected variables. To support input and output for the above services, *FetchDataHttp* downloads data from a web accessible folder or simply a URL to the WEE. *PublishDataWaf publishes* the data in the WEE to a web accessible folder.

**Fig 10:**
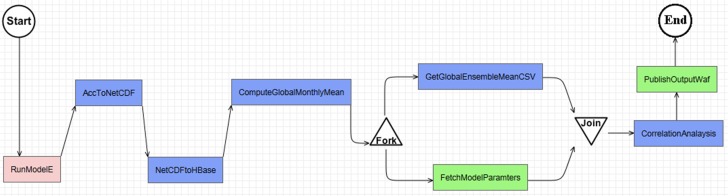
Executable workflow for the study case built in the prototype.

**Fig 11:**
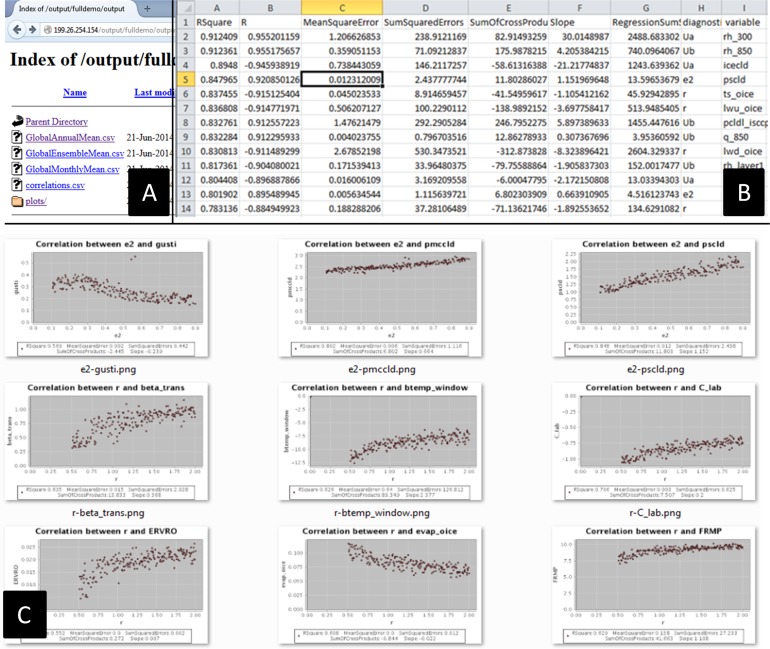
Workflow output: Workflow output published in a web accessible folder (A), presented as correlation statistics in CSV format (B), and plotted output climate variables highly affected by the seven model input parameters (R^2^ > 0.6, 9 of 57 pairs).

Once these services are registered, an executable workflow is built by visually dragging and connecting services in the *Web-based Workflow Builder* to conduct the experiment (Fig. 10). In this workflow, *RunModelE* provisions virtual machines to run the climate model. When model runs are finished, output are preprocessed and loaded to HBase with *ArcToNetCDF* and *NetCDFtoHBase*. Then global monthly mean for each output climate variable is calculated in parallel in the WEE with *ComputeGlobalMonthlyMean* service. Next, two services *GetGlobalEesembleMean* and *FetchModelParamters*, are executed in parallel. Once finished, *CorrelationAnalysis* service calculates linear regression statistics for each Parameter-Variable pair based on the variable ensemble mean values and the model input parameters. Finally, the workflow output (intermediate and final) is published on a web accessible folder (Fig. 11).

**Fig 12:**
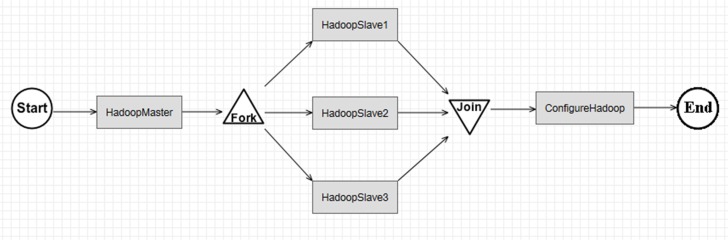
An executable workflow for provisioning a Hadoop cluster on-demand in the cloud. Three Infrastructure Services support this provision: ProvisionMaster, ProvisonSlave, and ConfigureHadoop.

This workflow transforms a complex geoscience experiment into an intuitive diagram-based workflow. In contrast to a traditional workflow, this workflow addresses the three problems of data intensity, computing intensity, and procedure complexity as presented below:

- For the computing intensity, *RunModelE* service on-demand provisions a cluster of virtual machines with pre-configured model environment and on-demand parameter configuration to conduct ensemble model runs in parallel. In addition, a Hadoop cluster is provisioned on-demand in the workflow (Fig. 12);
- For data intensity, the MapReduce-enabled *ComputeGlobalMonthlyMean* service conducts parallel processing of large volumes of model output in the cloud-based WEE; and
- For the procedure complexity, the service model enables the complex problem to be decoupled into reusable services. Furthermore, the heterogeneous software components (e.g., Hadoop, R, NCO, JRE) are seamlessly integrated in the cloud-based WEE.

#### 4.2.2. Performance Evaluation for Big Climate Data Processing

**Fig 13:**
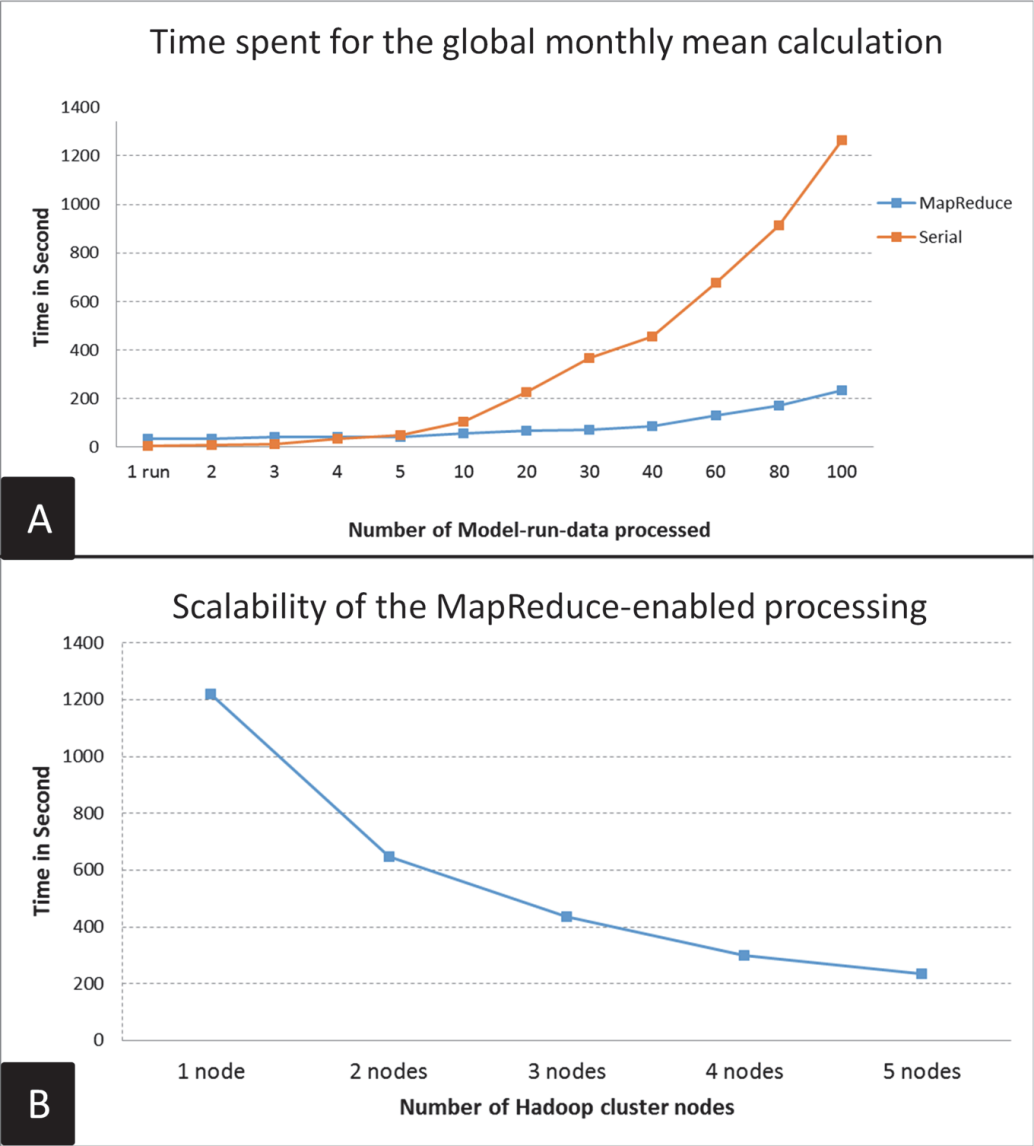
Performance evaluation result for the MapReduce-enabled big climate data processing.

To evaluate the performance of the big geoscience data processing strategy (Section 3.2), we calculated the global monthly mean for 100 model outputs using a 6-node Hadoop cluster (1 master node and 5 slave nodes). Each node is a virtual machine with 8-core CPU/2.35 GHz with 16 GB RAM, and the 100 model outputs are preprocessed and loaded to HBase deployed on the Hadoop cluster. Another virtual machine with the same configuration processes the same data with the traditional serial method. Two sets of tests are conducted. The first keeps the number of cluster nodes the same and processes different numbers of model outputs from 1 to 100 (Fig. 13A). The second keeps the 100 model output unchanged but changes the number of cluster nodes from 1 to 5 (Fig. 13B).

For the first set of tests and as model output number increases, the time consumed for the serial method increases dramatically for 5 model outputs, whereas the time for MapReduce approach only increases marginally (give a percentage) (Fig. 13A). With 100 outputs, the serial process takes > 20 minutes, while the MapReduce approach takes ∼3.5 minutes. It should be noted that if the number of model output is < 5, the time for the MapReduce approach is more than that of the serial approach due to the overhead of the Hadoop framework. For the second set of tests and with increasing node number, the consumed time decreases significantly (Fig. 13B), which indicates efficient scalability of the proposed big geoscience data processing strategy. Scalability is important in cloud environment because new nodes are quickly provisioned and added to the cluster as needed to improve performance.

## Discussion and Conclusion

This paper proposes a cloud-based, MapReduce-enabled, and service-oriented workflow framework to address the challenges posed by big geoscience data analytics. These challenges are tested by a case study of climate model sensitivity diagnostics. Methodologies for designing and implementing the framework are presented. To test the feasibility of the framework, a proof-of-concept workflow platform prototype is offered. A set of services are developed and registered to the prototype system, and an executable workflow is built based on these services for the study case. Two sets of tests are conducted to evaluate the performance of the proposed big geoscience data processing strategy.

The workflow and test results show that the proposed framework provides a robust and efficient approach to accelerate geoscience studies. Each proposed methodology addresses one or several aspects of the challenges facing the geosciences community. Specifically, Table 2 summarizes the proposed methodologies (Section 3) for addressing the corresponding challenges (Section 1).

**Table 2:** Methodologies addressing the challenges.

**Challenges**		**3.1**	**3.2.1**	**3.2.2**	**3.3.1**	**3.3.2**	**3.4**
C1. Data intensity	Data management	x	x				
	Distributed data	x				x	
C2. Computing intensity	Model simulation	x			x		x
	Big data processing	x	x	x			x
C3. Complex procedure	Complex steps	x			x		
	Heterogeneous tools	x			x		x

By leveraging cloud computing, MapReduce, and SOA, this framework seamlessly integrates the proposed methodologies as a whole to form a scalable, reliable and interoperable workflow environment. Such a workflow environment enables scientists to achieve four goals: transform complex geoscience experiment into intuitive diagram-based workflows by decoupling the experiment into reusable services; manage big geoscience data in a scalable and reliable distributed environment; process big geoscience data in parallel by adapting MapReduce and provide on-demand; and provision computing resources during the workflow execution to meet the performance requirement.

### 5.1. Key Features of the Workflow Framework

This framework provides three features compared to traditional scientific workflow platform as presented below:

- **Cloud-based for computing intensity:** Adequate computing resources are critical since scientific workflow normally contains computing intensive tasks and require hundreds of steps executed in parallel. This workflow framework provides the mechanism to supply adequate computing resources to the WEE by provisioning more VMs into the WEE and shifting the computing load to resources independent of WEE using *Infrastructure Services* (e.g., running a computing intensive model on a virtual machine). In addition, the entire WEE is provisioned based on customized VM images, and virtualization enables each node of the WEE to have exactly the same computational environment. Therefore, this framework provides provenance for the WEE in a bitwise level. This cloud-based feature helps address computing intensity challenges.
- **MapReduce-enabled for data intensity:** By incorporating the big geoscience data processing strategy (Section 3.2), the proposed framework manages and processes big geoscience data. The data decomposition and storage mechanism enables the multi-dimensional geoscience data to be effectively stored in a distributed environment (HBase), while the MapReduce-enabled processing framework enables data to be processed in parallel on the cluster of WEE chained with other tasks in the workflow. The MapReduce-enabled feature helps address the data intensity challenge.
- **Service-oriented for procedure complexity**: Different steps involved in scientific workflows are published as four types of services: process, data, model and infrastructure (Section 3.3). In contrast to traditional scientific workflow considering only computational tasks, infrastructure services enable scientists to provision on-demand more computing resources during the workflow execution. Model services enable scientists to integrate an entire modeling environment to the workflow. By introducing a unified service model, these services are registered to the framework and connected in a unified manner. In addition, the service-oriented mechanism opens the framework, allowing scientists to collaborate by publishing their own services and workflows. Thus, the service-oriented feature helps address the challenge of procedure complexity.

### 5.2. Future Research

As a preliminary study, this framework has limitations. There are at least two major challenges that need to be addressed in the future:

- The framework for data storage currently uses virtual storage attached to the VMs to form the HDFS. The storage attached to each VM is of two types. The first is virtualized directly from the physical machine on which the VM is hosted, and the stored data are accessible directly by the VM without going through any network. However, such storage is not permanent, and the data are lost with the termination of the VM. The second storage type is virtualized from a storage cluster connected to the cloud platform and persists even when the VM is terminated. However, since the storage is from a storage cluster instead of the VM’s host machine, the VM needs to access the data remotely. Therefore, neither storage type is optimized for the framework. Further study is desired to explore a new storage mechanism to support both local access and persistence.
- We only consider the private cloud in the prototype system. While a private cloud may be enough for a research center, spike workload normally cannot be handled due to the limited resources. To address this problem, a hybrid cloud mechanism is a candidate for the framework, using full-controlled private cloud as the primary cloud while bursting to the public cloud (e.g., Amazon EC2) for extra computing resources when needed.

Data intensity, computing intensity and procedure complexity are grand challenges in the geosciences even with 21^st^ century computing technologies. The proposed framework offers a potential solution to solve these challenges. This framework serves as a path to a common geospatial cyberinfrastructure platform shared by the geoscience community to relieve scientist from computing issues and facilitate scientific discoveries.
